# Tricarboxylic Acid Cycle Regulation of Metabolic Program, Redox System, and Epigenetic Remodeling for Bone Health and Disease

**DOI:** 10.3390/antiox13040470

**Published:** 2024-04-17

**Authors:** Wei-Shiung Lian, Re-Wen Wu, Yu-Han Lin, Yu-Shan Chen, Holger Jahr, Feng-Sheng Wang

**Affiliations:** 1 Core Laboratory for Phenomics and Diagnostic, College of Medicine, Chang Gung University, Kaohsiung Chang Gung Memorial Hospital, Kaohsiung 833401, Taiwan; lianws@cgmh.org.tw (W.-S.L.); ggyy5872024@cgmh.org.tw (Y.-S.C.); 2 Center for Mitochondrial Research and Medicine, College of Medicine, Chang Gung University, Kaohsiung Chang Gung Memorial Hospital, Kaohsiung 833401, Taiwan; cgmhlinyh202@cgmh.org.tw; 3 Department of Medical Research, College of Medicine, Chang Gung University, Kaohsiung Chang Gung Memorial Hospital, Kaohsiung 833401, Taiwan; 4 Department of Orthopedic Surgery, College of Medicine, Chang Gung University, Kaohsiung Chang Gung Memorial Hospital, Kaohsiung 83301, Taiwan; ray4595@cgmh.org.tw; 5 Department of Anatomy and Cell Biology, University Hospital RWTH, 52074 Aachen, Germany; hjahr@ukaachen.de; 6 Department of Orthopedic Surgery, Maastricht University Medical Center, 6229 HX Maastricht, The Netherlands

**Keywords:** osteoporosis, bone homeostasis, TCA cycle, redox, α-ketoglutarate, histone demethylases, DNA demethylases, RNA m6A demethylases

## Abstract

Imbalanced osteogenic cell-mediated bone gain and osteoclastic remodeling accelerates the development of osteoporosis, which is the leading risk factor of disability in the elderly. Harmonizing the metabolic actions of bone-making cells and bone resorbing cells to the mineralized matrix network is required to maintain bone mass homeostasis. The tricarboxylic acid (TCA) cycle in mitochondria is a crucial process for cellular energy production and redox homeostasis. The canonical actions of TCA cycle enzymes and intermediates are indispensable in oxidative phosphorylation and adenosine triphosphate (ATP) biosynthesis for osteogenic differentiation and osteoclast formation. Knockout mouse models identify these enzymes’ roles in bone mass and microarchitecture. In the noncanonical processes, the metabolites as a co-factor or a substrate involve epigenetic modification, including histone acetyltransferases, DNA demethylases, RNA m6A demethylases, and histone demethylases, which affect genomic stability or chromatin accessibility for cell metabolism and bone formation and resorption. The genetic manipulation of these epigenetic regulators or TCA cycle intermediate supplementation compromises age, estrogen deficiency, or inflammation-induced bone mass loss and microstructure deterioration. This review sheds light on the metabolic functions of the TCA cycle in terms of bone integrity and highlights the crosstalk of the TCA cycle and redox and epigenetic pathways in skeletal tissue metabolism and the intermediates as treatment options for delaying osteoporosis.

## 1. Introduction

Bone mass homeostasis is a dynamic process of mineralized component turnover balanced by osteoblast/osteocyte-mediated bone formation [1] and osteoclastic resorption [1]. Osteoblasts produce key osteoclastogenic factors, such as receptor activator of nuclear factor-kB ligand (RANKL), inflammatory cytokines or chemokines, for coupling osteoclastic cells for bone remodeling [2]. Osteocytes play an important role in sclerostin production and transmit biomechanical or biophysical stimulation for bone mass homeostasis [1]. Under menopause, aging, hyperglycemia, or chronic inflammation conditions, bone remodeling goes far beyond mineral acquisition, turning the well-arranged bone trabecular network into a porous microstructure to accelerate the development of osteoporosis [3]. The fragile biomechanical features put the osteoporotic skeleton under an incredibly high risk of fracture, accounting for the leading cause of skeletal disability associated with premature mortality in the geriatric population [4,5].

Plenty of bone anabolic factors, including vitamin D, parathyroid hormone (PTH), the Wnt/sclerostin pathway, bone morphogenetic proteins, and insulin-like growth factor 1, are advantageous to the bone-making capacity by increasing mineralized extracellular matrix buildup in osteogenic cell populations [6]. Osteoclast-regulatory cytokines, like interleukin-6, tumor necrosis factor-α, and RANKL together with reactive oxygen species (ROS) enhance the bone-resorbing activity of osteoclastic cells [7] during skeletal development, osteoporotic degeneration, or fracture healing [8]. A deeper insight into the latest metabolic mechanisms contributing to bone cell function prompts the development of treatment options for osteoporotic diseases.

Tricarboxylic acid (TCA) cycle metabolism is indispensable in the electron transfer chain [9] and redox homeostasis [10,11] in the mitochondrial microenvironment, maintaining the energy supply and intracellular signaling transduction in the physiological or pathological context. Key enzymes and intermediates of the TCA cycle exert canonical and noncanonical effects on bone-forming cells and osteoclasts, influencing bone physiology through biochemical and epigenetic pathways [12]. Plenty of in vivo models have shown that the genetic manipulation of these TCA cycle enzymes or biochemical control of the intermediates preserve bone tissue integrity, delaying degenerative bone or joint diseases [13].

Epigenetic dysregulation is correlated with the development of human and murine osteoporotic diseases and bone tumors [14]. For example, the hypermethylation of DNA or histones by specific methyltransferases represses epigenomic control of osteogenic activity or bone metabolism [15,16,17]. Furthermore, increasing evidence reveals that TCA cycle enzymes or intermediates, like isocitrate dehydrogenase (IDH), succinyl-CoA, acetyl-CoA, succinate, and α-ketoglutarate, are required by a plethora of epigenetic pathways, which are modified by histone acetyltransferases, DNA demethylases, RNA demethylases, or histone demethylases, changing chromatin accessibility for the transcription of gene targets [18] or lineage commitment of stem cells [19]. The interplay of the TCA cycle and epigenetic pathways for bone homeostasis warrants elaborate reviews.

This article sheds light on the canonical actions of TCA cycle enzymes and intermediates in terms of osteoblastic activity, osteoclast formation, and bone homeostasis and conveys new insight into the non-canonical role of these molecules in the epigenetic control of bone mass, as well as highlights the TCA metabolite treatment options for slowing down the development of osteoporotic skeletons.

## 2. The Function of TCA Cycle Metabolism in Bone Mass

The TCA cycle ultimately produces reduced forms of nicotinamide dinucleotide (NADH) and flavin adenine dinucleotide (FADH2) together with guanosine triphosphate (GTP) and CO_2_ by converting acetyl-CoA, which originates from carbohydrate, protein, or lipid metabolism. Eight crucial enzymes and nine intermediates are involved in these sophisticated biochemical reactions [20]. The TCA cycle is not only indispensable in the mitochondrial oxidative phosphorylation process for ATP production but is also important in catalyzing the biosynthesis of proteins, lipids, glucose, pyrimidine, and heme through anaplerotic reactions [21]. The use of ^13^C-tracing analysis and high-throughput ultrahigh-performance liquid chromatography–mass spectrometry for characterizing metabolomic landscapes and genetic or biochemical manipulation prompts us to understand the role each key enzyme or intermediate may play in bone cell function during skeletal tissue development and deterioration [22]. These enzymes or intermediates affect biological activities or metabolism in the bone microenvironment through canonical and non-canonical pathways.

### 2.1. Canonical Actions of TCA Cycle in Osteogenic or Osteoclastogenic Capacity

**Figure 1:**
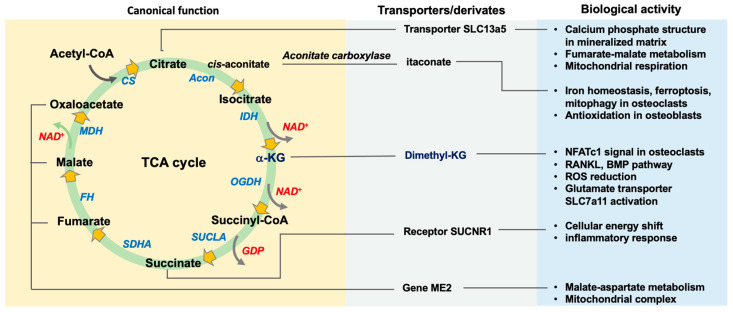
Schematic of the canonical functions of the TCA cycle in bone cell biology. Enzymes and intermediates affect a plethora of biological activities in bone-forming cells and bone-resorbing cells, at least, in part, through specific transporters or receptors. Derivates of intermediates modified by biochemical or organic processes exert regulatory effects on bone cells. Abbreviations: Acon, aconitase; IDH, isocitrate dehydrogenase; OGDH, oxoglutarate dehydrogenase; SUCLA, succinyl-CoA ligase; SDHA, succinate dehydrogenase A; FH, fumarate hydratase; MDH, malate dehydrogenase; CS, citrate synthase.

TCA cycle is crucial to electron transfer chain reaction in mitochondrial energy metabolism for growth and differentiation of bone cells. Gain or loss of function of the key enzymes impedes redox capacity, iron homeostasis, autophagy, and metabolic activity in bone cells. Gene knockout of the enzyme causes bone deformity. Decreased TCA cycle intermediate levels in serum are correlated with human and murine osteoporosis. These intermediates are key components in the formation of mineralized matrices of bone tissue; and change intracellular signaling transductions prompting bone-forming cells and osteoclastic cells to adapt hypoxic or inflammatory or oxidative stress conditions (Figure 1).

#### 2.1.1. Citrate, Citrate Synthase, and Acetyl-CoA

Citrate synthase converts oxaloacetate and acetyl-CoA into citrate, which accounts for 5% of the organic component of bone tissue that occupies approximately 80% of citrate in the body. An experimental mineralization process in bone cell models revealed that this intermediate is important in forming octacalcium phosphate and dicalcium phosphate dehydrate for mineralized matrix biosynthesis in osteoblasts incubated under an osteogenic condition [23]. Osteoblasts incorporate citrate through specific citrate transporter solute carrier family 13 member 5 (Slc13a5), which is required in osteoblast differentiation, mineralized nodule formation, and bone integrity. Osteoblast-specific Slc13a5 knockout mice develop a low trabecular bone microstructure and have decreased biomechanical strength. Slc13a loss inhibits malate and fumarate metabolism and mitochondrial respiration by enhancing Zip1 signaling [24]. Biophysical fluid flow promotes citrate production in osteocytes, while high glucose represses these effect [25]. Decreased citrate in bone tissue and plasma is correlated with human osteoporosis. Low serum citrate levels are also present in age-, ovariectomy- or excess retinoic acid-mediated osteoporosis in mice and rats [26]. Reduced citrate synthase levels and glycolytic capacity together with ATP underproduction are present in osteoblasts that are incubated with chondrocytes in a noncontact model [27]. Citrate synthase, the mitochondrial OXPHOS enzyme, and the antioxidants catalase, superoxide dismutase, and glutathione reductase decrease during heavy metal cadmium-mediated osteoblast dysfunction [28].

#### 2.1.2. Aconitase, Cis-Aconitate, and Itaconate

Aconitase modifies citrate into the intermediates cis-aconitate and isocitrate. This enzyme is important in osteoclastogenic capacity and the immune response of macrophages. It mediates RANKL-induced ferroptosis, ferritinophagy, and osteoclast formation of bone marrow macrophage progenitor cells [29]. The enzyme, in concert with iron regulatory proteins [30], controls iron homeostasis and hypoxia-inducible factor-1α signaling within the intracellular compartment, regulating autophagosome formation in a hypoxic environment [31].

In addition, cis-aconitate can be decarboxylated into itaconate by aconitate carboxylase. Itaconate in the mitochondrial compartment is transferred to the cytoplasmic compartment via ATP-binding cassette transporter [31]. This aconitate derivate has multiple functions, including anti-atherosclerosis [32], anti-inflammation [33], and anti-oxidation functions [34]. Decreased serum itaconate levels are correlated with estrogen deficiency-mediated bone loss. A role for Nrf2 in bone healing pathologies has been identified long ago [35]. Octyl itaconate promotes the Nrf2 signaling pathway, repressing oxidative stress and the RANKL-induced osteoclast formation of bone marrow macrophages [36]; it also enhances the Nrf2-mediated antioxidant response element, heme oxygenase, and NADPH quinone dehydrogenase 1, and the glutamate–cysteine catalytic subunit. The itaconate derivate also attenuates hydrogen peroxide-induced apoptosis and mineralized matrix loss in osteoblasts [37,38]

#### 2.1.3. Isocitrate Dehydrogenase and α-Ketoglutarate

Isocitrate is decarboxylated into α-ketoglutarate (α-KG) together with NADPH production by isocitrate dehydrogenase (IDH), which is a rate-limiting enzyme of the TCA cycle. However, what role this enzyme may play in skeletal tissue development remains uncertain. For example, a mice germline deficient in IDH2 developed typical signs of scoliosis, including spinal deformity, a porous trabecular bone network (decreased trabecular bone volume, thickness, and number), and decreased osteoclast number [39]. However, the study by Lee SH et al. on similar IDH2 knockout mice uncover increased bone mineral density and trabecular bone network and decreased osteoclastic erosion in distal femurs. The trabecular bone volume in the lumbar vertebrae of knockout mice was also higher than that in wild-type mice [40]. Loss of IDH2 function represses osteoclast formation of bone marrow macrophages by inhibiting the expression of the key osteoclastogenic cytokine RANKL in osteoblasts. IDH2 knockout compromises the loss in trabecular bone mineral density and volume and represses osteoclast number rather than osteoblast number in ovariectomized mice [40].

Increasing evidence has associated decreased serum α-KG levels with human osteoporosis. Long-term supplementation with α-KG counteracts age-mediated trabecular microstructure loss and reverses the growth and osteogenic differentiation capacity of bone marrow mesenchymal progenitor cells by enhancing BMP signaling. It also promotes the regeneration of femur defects in rats [41]. In addition to bone protection, this metabolite has chondroprotective effects on joints, as α-KG attenuates oxidative stress and the extracellular matrix underproduction of inflamed chondrocytes, preserving the mitophagic program to delay osteoarthritis development in destabilized medial meniscus-induced knee injury [42].

The effects of α-KG on osteoclast function appear to be context-dependent. For example, this TCA cycle metabolite mediates the serine metabolism pathway, enhancing the key osteoclastogenic transcription factor nuclear factor of activated T cells, cytoplasmic 1 (NFATc1) signaling, and osteoclastic activity [43]. However, Lee et al. reveal that α-KG mediates glutamate transporter solute carrier family 7 member 11 (Slc7a11) signaling, impeding osteoclast differentiation of RANKL-induced bone marrow macrophage precursor cells. TRAP-stained multinuclear osteoclast formation and resorbed pit formation are repressed upon incubation in α-KG. This metabolite also attenuates liposaccharide-mediated osteoclastic resorption in murine calvariae [44].

#### 2.1.4. Succinate and Succinate Dehydrogenase

α-KG is converted into succinyl-CoA and succinate together with NADP and GTP production by α-KG dehydrogenase and succinyl-CoA synthetase, respectively. Transferring defective mitochondria of bone marrow macrophages from ovariectomy or liposaccharide-mediated osteoporotic skeleton into mesenchymal progenitor cells causes a glycolysis/mitochondrial energy shift and succinate over-accumulation, which increases inflammatory cytokines to inhibit osteogenic marker expression and mineralized matrix synthesis [45]. Succinate enhances the inflammatory response by activating succinate receptor 1 (SUCNR1) in an experimental periodontitis model. Gene knockout or pharmacological antagonism of SUCNR1 counteracts periodontitis-induced bone mass loss, whereas intraperitoneal injection of succinate exacerbates periodontitis-mediated bone microstructure destruction [46]. Succinate dehydrogenase (SDH) is a crucial enzyme modifying succinate into fumarate. Whether these enzymes or intermediates influence bone-forming cells, osteoclasts or osteoporotic bone development remains uncertain.

#### 2.1.5. Fumarate, Malate, and Oxaloacetate

The production of malate, oxaloacetate, and NADP is catalyzed by fumarase and malate dehydrogenase. The expression of Me2, a gene that encodes malic enzyme 2, is increased during mineralized matrix biosynthesis in murine calvaria osteoblasts. The silencing of Me2 by RNA interference disrupts malate–aspartate metabolism, inhibiting glycolytic activity, i.e., key ATP production for osteoblastic activity [47]. Succinate and fumarate production interruption together with decreased mitochondrial complexes and malate dehydrogenase is present in osteoblasts upon incubation under a microgravity condition [48]. Dimethyl fumarate preserves the expression of key osteocyte markers, including dentin matrix protein and sclerostin [49]. Oxaloacetate administration accelerates skeletal tissue development in young mice, including increased trabecular thickness and bone mineral density. The growth and differentiation capacity of osteoblasts are also increased by this intermediate [50], while bone mass or microstructure in digits is unaffected in aged mice upon oxaloacetate treatment [51].

#### 2.1.6. Acetyl-CoA

Acetyl-CoA is a key substrate for citrate production catalyzed by citrate synthetase. This metabolite is converted by ATP-citrate lyase (ACLY). The reduction of acetyl-CoA by ACLY RNA interference or the inhibitor BMS-303141 represses the RANKL-mediated osteoclast formation of bone marrow macrophages. BMS-303141 administration counteracts the loss in trabecular and cortical bone microarchitecture, as well as the osteoblast surface, but attenuates osteoclast distribution in ovariectomized mice [52]. In bone-forming cells, acetyl-CoA is required to maintain the osteogenic lineage commitment of mesenchymal stem cells under hypoxic conditions. Normoxia causes acetyl-CoA accumulation in mitochondria by reducing citrate carrier activity, driving mesenchymal stem cell differentiation into adipocytes. Hypoxia reverses acetyl-CoA exportation, preserving the osteogenic differentiation capacity [53]. Acetyl-CoA produced from glutamine metabolism, which is stimulated by crucial chondrogenic transcription factor SRY-box transcription factor (Sox9), regulates the epigenetic pathway control of glutamate dehydrogenase, contributing to chondrogenic gene transcription in growth plate chondrocytes [54].

### 2.2. Glutamate Metabolic Control of the TCA Cycle

Glutamate is also an important substrate of α-KG synthesis catalyzed by glutamate dehydrogenase. A cohort study on the serum metabolomic profiles of 1193 females associates glutamate and TCA cycle metabolism with menopausal osteoporosis [55]. Glutamine is converted into glutamate by glutaminase. Mice deficient in glutaminase in osteogenic progenitor cells develop decreased cortical bone thickness, osteoblast distribution, and dynamic bone formation capacity, as well as α-KG underproduction together with decreased osteogenic activity of bone marrow mesenchymal cells compared to wild-type mice. α-KG treatment preserves the growth and differentiation of mesenchymal stem cells in glutaminase knockout mice [56]. Furthermore, glutamate supplement steers mesenchymal progenitor cells towards osteogenic lineages and improves the bone mass and microstructure in mice upon hindlimb unloading [57]. The effect of the glutaminase inhibitor CB-839 on bone turnover appears to be osteoporosis type-dependent. CB-839 counteracts trabecular bone loss, osteoclast overburden, and increased osteoclast formation of bone marrow macrophages in ovariectomized mice through metabolomic alterations contributing to the TCA cycle, amino acid, nucleotide, and glutathione oxidation metabolism in osteoclasts. However, the agent exacerbates the loss in the trabecular bone network, the osteoblast surface, and the mineralized matrix production of bone marrow mesenchymal cells in aged mice by influencing the amino acid, nucleotide, and glutathione metabolomes in osteoblasts [58].

In bone-resorbing cells, the loss of glutamate function by blocking glutamate transport or by incubating under a glutamate-free condition impairs osteoclast formation and pit formation of bone marrow macrophages, slowing down bone loss in ovariectomized mice. Glutamate enhances osteoclastic resorption and osteoporosis development by reducing α-KG production [59]. The glutamate-α-KG metabolism pathway also changes the osteoclastogenic cell fate. Extracellular vesicles produced by M2 macrophages shift osteclastogenic progenitor cells into M2 macrophages and reverse trabecular network loss and osteoclastic resorption in ovariectomized mice. These extracellular vesicles promote glutamate/glutamine metabolism and α-KG production, which reduces RANKL-mediated osteoclastogenic marker expression and osteoclast formation [60]. Additionally, branched-chain amino acids, like leucine and isoleucine, are involved in glutamate-α-KG metabolism for osteoclast formation through branched-chain aminotransferase. The loss of function of this enzyme by the inhibitor gabapentin or inactive mutation counteracts RANKL-mediated osteoclast formation and LPS-induced calvaria bone resorption [61].

Bioinformation (Table 1) reveals that each TCA cycle enzyme is found to interact with a plethora of genes, which contribute to multiple intracellular signaling pathways and metabolic activities, including glycolysis, mitochondrial complex assembly, OXPHOS for cellular energetics, amino acid metabolism, urea cycle metabolism, fatty acid biosynthesis, and glutathione metabolism. Of note, α-KG dehydrogenase may affect VEGF–VEGFR2 and the angiogenesis pathway. Aconitase, IDH, or citrate synthetase influences cytosine methylation, histone acetylation, or histone methylation. The gene–gene interaction suggests that noncanonical pathways may be regulated by the TCA cycle.

## 3. The TCA Cycle Regulates Redox Homeostasis

The TCA cycle produces NADH and NADPH as co-factors, which are important to redox homeostasis in the oxidative phosphorylation process through electron transfer chain (ETC) complexes for ATP synthesis. Electrons in cytochrome c of Complex IV are mainly converted into O_2_, whereas very few electrons are leaked from Complex II or Complex III to produce superoxide, hydroxyl radical, and hydrogen peroxide [62]. The dysregulation of TCA cycle enzymes or intermediates enhances oxidative stress, which causes macromolecule instability, cell apoptosis or tissue dysfunction.

Aconitase together with frataxin forms a [4Fe–4S]^2+^ cluster as the redox sensor interacts with plenty of reactive oxygen radicals, including H_2_O_2_, ONOO^−^, and O_2_^−^, turning mitochondrial [4Fe–4S]^2+^ into the [3Fe–4S]^+^ cluster during aging or mitochondrial dysfunction [63]. α-KG dehydrogenase and pyruvate dehydrogenase account for a large amount of H_2_O_2_ production in mitochondria. The disruption of these enzymes causes H_2_O_2_ overproduction, repressing mitochondrial metabolism [64]. IDH loss interrupts GSH and NADPH homeostasis, worsening high-fat diet-induced mitochondrial reactive oxygen species production and oxidative damage [65], and enhances mitochondrial NADP+/NADPH and NAD+/NADH underproduction, as well as the loss of the antioxidants superoxide dismutase and catalase [66]. Reactive oxygen radicals are increased in the hepatic, cardiac, renal, and muscle tissue of IDH knockout mice [39]. Metabolomic landscapes have revealed that the inhibition of the TCA cycle by fumarate hydratase enhances antioxidant glutathione accumulation together with amino acid dysmetabolism through impairing mitochondrial thiol metabolism [67]. Malate dehydrogenase and succinate dehydrogenase are indispensable in preserving NAD+ production and mitochondrial redox capacity for cell viability upon TCA cycle dysfunction [68].

A balanced redox capacity in mitochondria is required to maintain bone mass homeostasis; however, the loss of antioxidant function decreases bone-forming cell survival, reducing bone formation [69]. The overproduction of reactive oxygen radicals by aging or chronic inflammation promotes macrophage differentiation into osteoclastogenic lineages, increasing the bone resorption capacity and accelerating bone remodeling [70]. While oxidative stress is present in the development of age- or estrogen deficiency-mediated osteoporosis, which may be directly regulated through SOX9 [71], what the role the TCA cycle enzymes or intermediates may play in this feature warrants studies.

## 4. Noncanonical Actions of TCA Cycle Metabolism in Bone Formation and Resorption

**Figure 2:**
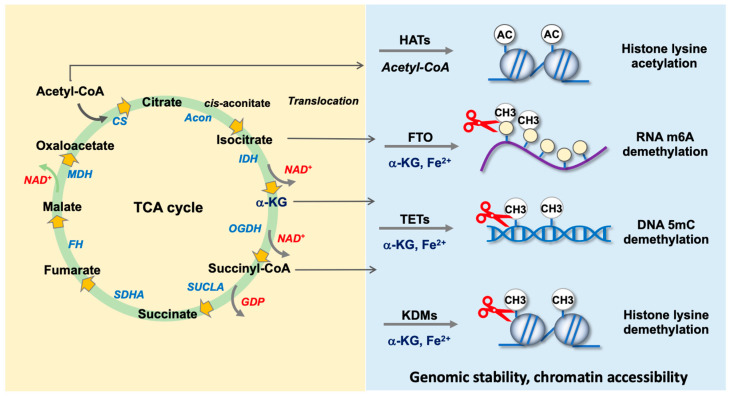
Schematic drawing for the noncanonical functions of the TCA cycle in bone cell biology. IDH translocates into the nuclear compartment. The intermediates acetyl-CoA is a substrate of histone acetyltransferase for catalyzing histone acetylation. α-KG is a co-factor of DNA, RNA or histone demethylases for removing methyl groups. Succinyl-CoA involves histone succinylation. Abbreviations: HATs, histone acetyltransferases; FTO, fat mass and obesity-associated protein; TETs, tet methylcytosine dioxygenase; KDMs, histone lysine demethylases; m6A, methylated N^6^-methyladenonsine; 5mC, 5-methylcytosine; Ac, acetyl; CH3, methyl.

TCA cycle metabolism not only contributes to mitochondrial energy metabolism and redox balance but also plays an important role in the epigenetic pathway control of cell function [72]. Contemporary studies have uncovered the translocation of TCA cycle enzymes into the nucleus compartment [73] and the co-factor function of TCA cycle intermediates for enzymatic modification of DNA, RNA or histones for genome stability and chromatin accessibility, which affect the transcriptome and epigenome and then cell fate and metabolism [74]. Among metabolites, acetyl-CoA and α-KG are an indispensable substrate and co-factor, respectively, for plenty of epigenetic regulators (Figure 2).

### 4.1. Nuclear Translocation of TCA Cycle Enzymes

Kafkia et al. conduct ^13^C-tracer analysis and proximity labelling mass spectrometry, identifying a plethora of TCA cycle routes, including glutamate–fumarate and citrate–succinate metabolism together with key enzymes present in the nucleus. The nuclear α-KG dehydrogenase produces succinyl-CoA, which promotes the succinylation of nuclear components to maintain the stemness of embryonic stem cells [75]. Plenty of key TCA cycle enzymes, including pyruvate dehydrogenase, aconitase, citrate synthase, and isocitrate dehydrogenase, translocate into the nuclei of pluripotent stem cells. The genetic mutation of these enzymes affects stem cell reprogramming. Pyruvate dehydrogenase controls nuclear acetyl-CoA production, which promotes the acetylation of lysine 3 and lysine 27 at histone 3 [75]. The inhibition of mitochondrial complex III by antimycin A promotes osteogenic differentiation, chondrocyte development, and adipocyte formation of skeletal mesenchymal progenitor cells. This biochemical manipulation prompts stem cells to develop into ectopic bone tissue in mice. The succinate dehydrogenase-α-KG pathway in the nucleus reduces CpG methylation in DNA to maintain the lineage specification capacity by enhancing DNA demethylase activity [76]. Little is known about how TCA cycle enzymes translocate into the nuclear compartment to interact with DNA or chromatin. The molecular mechanism contributing to the nuclear translocation of mitochondrial TCA cycle enzymes warrants characterization.

### 4.2. TCA Cycle Intermediates as Co-Factors for Epigenetic Regulators

In epigenetic pathways, the methylation of DNA and histones causes transcription repression of gene targets. The methyl modification of nucleotides in DNA and lysine in histones is catalyzed by specific DNA methyltransferases and histone methyltransferases, respectively. DNA and histone demethylases remove those methylated groups [77]. On the other hand, the acetylation of histone keeps chromatin in a loosen form, facilitating histone enrichment at the promoter region of the gene target [78]. The TCA cycle intermediate α-KG as a co-factor in concert with ferrous ions involves the demethylation reaction catalyzed by plenty of demethylases, including DNA demethylase tet methylcytosine dioxygenases (TETs) [79], lysin-specific histone demethylases (KDMs) [80], and RNA m6A demethylase fat mass and obesity-associated proteins (FTOs) [81]. Additionally, acetyl-CoA as a substrate for the acetylation of lysine in histones is catalyzed by histone acetyltransferases that change the chromatin configuration [82], and succinyl-CoA is a specific non-acetyl-CoA substrate of histone acetyltransferase KAT2 for catalyzing the succinylation reaction to histones [83]. Furthermore, increasing evidence has revealed that the inhibition of ATP-citrate lyase reduces nucleocytosolic acetyl-CoA production, repressing H3 acetylation to counteract RANKL-mediated osteoclast formation and bone loss [52]. M2 macrophage-derived exosomes promote glutamate metabolism and α-KG production, which enhances histone demethylase Jmjd3 function to reduce osteoclast formation [58]. Serine metabolism produces α-KG, affecting histone methylation for osteoclastogenic differentiation of bone marrow macrophages [43]. α-KG promotes the demethylation of H3K9, which is enriched at the Nrf2 promoter, inhibiting osteoclast formation [44]. The roles of histone acetyltransferases, DNA demethylases, RNA demethylases, and histone demethylases in bone mass are as follows.

#### 4.2.1. Acetyl-CoA as a Co-Factor for Histone Acetyltransferases

In histone acetylation, the acetyl group of acetyl-CoA is transferred to the ε-amino group of lysine in histones by histone acetyltransferases (HATs) as a “transcription writer” for chromatin accessibility [82]. Among HATs, CREB-binding proteins (CREBBPs) and p300 are required in bone tissue development. Osteoblast-specific CREBBP or p300 knockout mice develop low bone mass, trabecular volume, and biomechanical strength. Osteoporosis signs are present in mice upon p300 inhibitor treatment. These two HATs acetylate lysine 27 of histone 3, enhancing β-catenin signaling to promote osteoblastic activity [84]. This HAT interacts with nuclear receptor corepressor and histone deacetylase 3, promoting acetyl histone enrichment at toll-like receptor 4/6 promoters for osteoclast differentiation of macrophages [85]. Furthermore, single-cell sequencing has shown that p300 appears to be an osteoclast fate “decision maker”, which interacts with Glu/Asp-rich carboxy-terminal domain 2 (Cited2) to induce osteoclast maturation [86]. KAT2 acetylates H3K9, contributing to craniofacial structure development. It also regulates chondrocyte maturation by nonhistone acetylation of Raptor to enhance mTOR1 activity [87]. KAT8 acetylates H4K16 during osteoblast differentiation. The inhibition of KAT8 by MG149 represses H4K16ac binding promoters for osteogenic transcription factors Runx2 and Osterix, reducing osteogenic activity [88]. DNA methylome landscapes have revealed the hypermethylation of the KAT6B promoter region in patients with congenital scoliosis. The forced expression of KAT6B promotes the proliferation and expression of hypertrophic chondrocyte markers, including Runx2, Col10a1, VEGF, and β-catenin, in facet joint chondrocytes from patients [89].

#### 4.2.2. α-KG Is Required by DNA Demethylase TETs

TETs in concert with cofactors α-KG and Fe^2+^ demethylate 5-methylcytosine (5mdC) of DNA into 5-hydroxymethylcytosine (5hmdC), regulating bone tissue integrity. Mesenchymal stem cell-specific TET1, TET2, or TET3 knockout mice develop cleidocranial dysplasia and long bone underdevelopment together with decreased bone formation capacity of bone marrow mesenchymal stem cells. TETs enhance chromatin accessibility to a plethora of genes, contributing to osteogenic differentiation, as well as promote osteogenic transcription factor Runx2 function for osteogenic activity [90,91]. Increased TET function and DNA hypomethylation, which influences plenty of bone-regulatory genes, including Bmp2, Sp7, Dlx3, Dkk1, and Sost, are present in the vitamin C promotion of the osteogenic activity of bone marrow mesenchymal progenitor cells. L-2-hydroxyglutarate, an α-KG competitive inhibitor, represses 5hmC production in vitamin C-treated osteoblasts. Loss of TET2 function counteracts osteogenic gene expression and mineralized matrix formation in vitamin C-treated osteoblasts. Forced TET2 expression increases 5hmC levels and mineralized matrix production. TET1/2 knockout mice have a low trabecular bone volume [92].

The pharmacological inhibition of TET2 activity by Bobcat339 increases 5mC levels and reduces TET enrichment at the promoter of transcription factor Sp7, repressing alkaline phosphatase activity and Alizarin red-stained mineralized matrix synthesis of C2C12 mesenchymal progenitor cells. Gain of TET2 function counteracts Bobcat339-induced dysfunction of the stem cells [93]. Cakouros et al. reveal that TET1 and TET2 exert different effects on the lineage specification of mesenchymal stem cells. TET1 represses osteogenic differentiation and adipocyte formation by controlling histone methyltransferase EZH2 and SIN3A, whereas TET2 promotes both the osteogenic differentiation and adipocyte formation capacity of bone marrow mesenchymal stem cells [94].

Similar to the promoting effects of TET2 on osteogenic cells and bone formation, this molecule appears to be advantageous to osteoclastic activity. Loss of TET2 function by RNA interference reduces the autophagic program, osteoclast formation, and bone erosion capacity of RANKL-treated bone marrow macrophage precursor cells by enhancing Bcl2 signaling. TET2 interference by lentivirus shuttle RNAi mitigates trabecular bone loss and osteoclastic erosion in ovariectomized mice [95].

#### 4.2.3. α-KG as a Co-Factor for RNA m6A Demethylases FTO and ALKBH5

The methylation of N6-methyladenonsine in RNA is catalyzed by specific RNA methyltransferases as “writers”, like the methyltransferase-like protein (METTLE) family, which influence RNA processes. ALKBH5 and FTO, as an “eraser”, are α-KG and Fe^2+^-dependent m6A demethylases that remove the methyl group of adenosine [96].

Decreased FTO expression is correlated with the development of human osteoporosis and osteonecrosis. Loss of FTO function by RNA interference or putative inhibitor FB23 impedes the osteogenic differentiation of bone marrow stromal cells by regulating PPARγ signaling [97]. Zhang et al. demonstrate increased FTO expression in bone marrow stromal cells in patients with osteoporosis. FTO in bone tissue is required to prevent osteoblast dysfunction caused by extracellular stress. Ubiquitous FTO knockout mice develop a relatively small build together with decreased trabecular bone microarchitecture. Bone microstructure is unaffected in young osteoblast-specific FTO knockout mice (12 weeks old), whereas old knockout mice (30 weeks old) have evident bone loss, marrow adiposis, and a reduced bone formation rate. Osteoblasts from FTO knockout mice are more susceptible to high-energy irradiation or high-fat diet-induced DNA damage and apoptosis than wild-type mice by repressing the Hspa1a and NFkB pathways. Loss of FTO function enhances the methylation of m6A in Hspa1a [98].

FTO expression in bone marrow macrophages is increased in the osteoporotic bone of ovariectomized mice. Forced FTO expression increases osteoclast formation and the actin ring formation of RANKL-treated macrophages by regulating the NFkB binding osteoclastogenic transcription factor NFATc1 promoter. Silencing of FTO counteracts trabecular bone mass loss, osteoclastic erosion surface, and the osteoclast formation of bone marrow macrophage precursor cells in ovariectomized mice [99].

The role of ALKBH5 in osteogenic activity or osteoporosis development remains uncertain. Mesenchymal progenitor cell-specific ALKBH5 knockout mice have a relatively high bone mass and trabecular bone network. ALKBH5 knockdown enhances mineralized matrix production, and forced enzyme expression reduces the osteogenic activity of bone marrow mesenchymal cells by regulating the PRMT6-PI3K/AKT pathway [100]. However, Feng et al. demonstrate that the loss of ALKBH5 impairs the osteogenic differentiation of mesenchymal progenitor cells by reducing Runx2 stability [101].

#### 4.2.4. α-KG Is Indispensable in Histone Demethylase-Mediated Histone Demethylation

The Jumonji domain-containing (JMJD) protein histone demethylases, including the KDM4, KDM5, KDM6, and KDM7 families and JMJD6, are α-KG- and ferrous-dependent enzymes for the post-translational demethylation of the di- or trimethyl group of lysine in histones. Increasing evidence has uncovered these molecules’ role in osteogenic differentiation or osteoclastic activity. The JMJD family members are also found to regulate joint integrity.

#### 4.2.5. KDM4A and KDM4B

The forced expression of KDM4A enhances adipocyte formation by increased CEBPα and sFRP4 signaling; however, the loss of KDM4A function by mutation of this enzyme’s catalytic site promotes osteogenic differentiation of bone marrow mesenchymal cells. This enzyme reduces H3K9me3 levels and binds the promoter regions of adipogenic regulators CEBPα and sFRP4. It also induces non-histone methylation because forced expression of the enzyme represses the methylation of the CEBPα and sFRP4 promoters [102]. Body weight or long bone microstructure is unchanged in young mesenchymal stem cell-specific KDM4B knockout mice (3 months old). Aged knockout mice (12 or 18 months old) develop low trabecular and cortical bone microarchitecture and evident marrow fat formation. KDM4A loss worsens estrogen deficiency or high-fat diet-mediated bone loss and marrow adiposis. KDM4B deletion accelerates osteogenic cell senescence and counteracts PTH-mediated bone anabolism. The enzyme removes H3K9me3 marks and affects transcriptomic profiles contributing to OXPHOS and DNA replication together with H3K9me3 binding epigenomic landscape alteration, including Wnt and Runx2 [103].

This enzyme seems to exert inhibitory actions against osteoclastic resorption. Macrophage-specific KDM4B knockout mice develop a relatively high trabecular bone volume together with a lower osteoclast surface and osteoclast formation of bone marrow macrophage progenitor cells than wild-type mice. KDM4B, CCAR1, and MED1 form an interacting complex regulating H3K9 binding to the Fosl2 and Tpm1 promoters, which contributes to osteoclastogenic differentiation. The KDM4B inhibitor M324 reverses estrogen deficiency-mediated loss in trabecular bone volume, as well as bone mineral density [104].

#### 4.2.6. KDM5B and KDM5C

A whole genome sequencing study on UK Biobank participants has associated KDM5B, among others, with bone homeostasis [105]. Increased KDM5A levels are correlated with human and murine osteoporosis. Forced enzyme expression reduces H3K4me3 levels and the mineralized extracellular matrix production of BMP-2-treated mesenchymal stem cells; however, the methyl histone enrichment at Runx2 is elevated in these cells. KDM5A inhibitor administration attenuates bone loss in ovariectomized mice [106]. In osteoclastogenic cell-specific KDM5C knockout mice, the enzyme’s function in bone tissue is gender-dependent. Female knockout mice develop a denser trabecular network than wild-type mice, whereas the bone microstructure is unaffected in male knockout mice relative to gender-matched wild-type animals. KDM5C deletion represses glycolysis, mitochondrial respiration, OXPHOS, and osteoclast formation of bone marrow macrophage progenitor cells by disrupting PGC-1α signaling [107].

#### 4.2.7. KDM6A and KDM6B

Mice carrying the EZH2 R684C mutant, an experimental Weaver syndrome characterized by bone overgrowth, develop a relatively thick cortical bone, increased bone mineral acquisition, and mineralized extracellular matrix production of bone marrow stromal cells. The KDM6A/B inhibitor GSK-J4 counteracts bone overdevelopment in these genetically modified animals [108]. The deletion of KDM6A in cranial cells represses suture development and osteoblastic activity, including Runx2 and alkaline phosphatase expression of calvaria osteoblasts in female mice. Osteoblast differentiation capacity is enhanced in male knockout mice, and increased KDM6B function appears to compensate these effects [109]. KDM6B mediates the osteogenic differentiation of human mesenchymal stem cells. It reduces the H3K27me3 binding promoter regions of BMP2 and HOX6, which is indispensable in estrogen-mediated osteogenesis [110]. Furthermore, KDM6B involves the H3K27me3 binding mitochondrial transcription factor Tfam promoter in probiotic protection of bone mass loss and body adiposis in mice fed a high-fat diet [111].

#### 4.2.8. KDM7A

Osteoprogenitor cell-specific KDM7A knockout mice have a high trabecular volume and bone formation rate together with decreased osteoclast burden and marrow adipocytes. KDM7A loss drives osteoprogenitor cells away from adipocytes and represses osteoclast formation by enhancing H3K9me2 and H3K27me2 enrichment at the RANKL promoter to reduce osteoclastogenic cytokine expression for osteoclast formation. The ovariectomized knockout mice have a less serious osteoporosis condition than ovariectomized wild-type animals [112]. In addition to bone integrity, the KDM2/7 inhibitor daminozide promotes the extracellular matrix production of chondrocytes from human osteoarthritis biopsies by elevating H3K79 methylation and delays the development of osteoarthritis in destabilized medial meniscus-injured joints [113].

A bioinformation search (http://cytoscape.org) (Table 2) reveal that genes and biological activities may be regulated by epigenetic regulators. For example, FTO is found to interact with plenty of genes, affecting AMPK, SREBP, and the microRNA control of the YAP pathway and p53 pathway. KDMs influence DNA damage, nucleic acid metabolism, innate immunity, the circadian rhythm pathway, melatonin metabolism, the cell cycle, the vitamin D/calcium signaling pathway, androgen receptor signaling, and HIF-1α signaling, etc. Each histone demethylase regulates different histone-binding epitranscriptomic landscapes, which may contribute to cell growth, differentiation, or metabolism. The interplay of the TCA cycle and epigenetic regulators explains the natural complex of metabolic mechanisms contributing to bone homeostasis and osteoporosis development.

## 5. TCA Cycle Metabolites for Counteracting Osteoporotic Disorders

Based on in vitro and in vivo studies, the genetic or biochemical manipulation of TCA cycle metabolism may be advantageous for bone formation or resorption for controlling bone mass homeosis or the regeneration capacity of TCA cycle metabolites or derivatives; α-KG or the aconitate derivative itaconate has bone-protective actions.

### 5.1. Perspectives of Remedial Options

Supplementation with 0.25% or 0.75% α-KG in drinking water for 1 month increases serum α-KG levels and delays bone loss in aged mice. In addition, 0.75% α-KG supplementation for 1 month also accelerates bone regeneration in drilling-induced femur defects in rats [41]. Increasing studies have invented biomaterials for bone-targeting delivery of α-KG to enhance this intermediate’s therapeutic efficacy in slowing down osteoporotic disorders. For example, near-infrared emissive lanthanide luminescence nanoparticles have been developed to carry a very small amount of α-KG specifically into the bone tissue microenvironment. Trabecular bone loss and osteoclast overburden in ovariectomized mice are improved upon administration of these α-KG-carrying nanoparticles [114]. Dimethyl-α-KG is produced by chemically modifying α-KG into a membrane-permeable derivative, which cleaves into α-KG in the cytoplasm. Collagen gelfoam loaded with dimethyl-α-KG and BMP2 recombinant protein accelerate ectopic bone formation in young and aged mice, as compared to the gelfoam loaded with BMP2 only [115]. In addition, supplementation with 2% α-KG in drinking water for 8 weeks slows the development of osteoarthritis, including articular cartilage erosion and synovial hyperplasia in mice with destabilized medial meniscus-induced knee injury [42].

Intraperitoneal injection of 50 mg/kg 4-octyl itaconate, a derivate of itaconate, twice per week for 6 consecutive weeks attenuates the repression of the trabecular bone volume, thickness, and number together with decreased osteoclast number in ovariectomized mice [36]. Treatment with a Zn-based metal-organic supercontainer encapsulating 4-octyl itaconate counteracts oxidative stress, synovitis, and subchondral bone destruction in rats with inflammatory arthritis [116]. Intra-articular injection of 4-octyl-itaconate reverses articular cartilage erosion, synovial hyperplasia, and subchondral bone microstructure damage in mice with posttrauma-induced knee joint injury [117]. Dimethyl furamate compromises the loss in bone mass and trabecular volume in ovariectomized mice by reversing osteoclastic activity [49].

### 5.2. Limitations of TCA Cycle Metabolites for Slowing Osteoporosis

While plenty of proof-of-concept studies have revealed the remedial effects of α-KG and itaconate in experimental osteoporosis or knee osteoarthritis models, little is understood about whether these TCA cycle metabolites affect human osteoporosis development. α-KG has been used as a dietary supplement; whether off-target use may induce a metabolite burden on the function of other tissues warrants evaluations. There is a need for thoughtful randomized, double-blinded, placebo control clinical trials to evaluate the effects of these metabolites or derivatives on patients with osteoporotic disorders or osteoarthritis.

## 6. Conclusions

**Figure 3:**
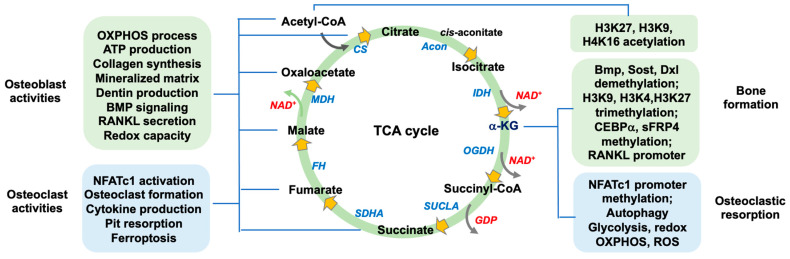
Schematic drawing of the TCA cycle regulation of osteoblast activity and osteoclast formation for bone formation and resorption.

This article conveys productive insight into the canonical and noncanonical actions of TCA cycle enzymes and intermediates in osteogenic activity and osteoclast formation during bone homeostasis and osteoporosis development. These molecules not only regulate mitochondrial energy metabolism and redox but also act as a co-factor or a substrate involve in epigenetic pathways by catalyzing the acetylation of histones and removing methyl groups in 5mdC in DNA, RNA m6A, and histones to change transcriptome or epigenome landscapes, which may contribute to bone formation or resorption (Figure 3). Studies on gene knockout mice have identified the regulatory roles of TCA cycle enzymes and DNA, RNA, and histone demethylases in bone tissue integrity. Whether these TCA cycle enzymes or epigenetic regulators are related to human osteoporotic diseases warrant more cohort studies.

Preclinical studies on animal models reveal that treatment with the TCA cycle intermediates α-KG, itaconate, and citrate and histone demethylase inhibitors are found to delay experimental osteoporosis or enhance bone regeneration in bone defects. Clinical trials are required to evaluate whether these compounds affect human osteoporotic diseases. This review illustrates the bone-regulatory actions of the TCA cycle in osteoblastic and osteoclastic activity by affecting mitochondrial energy biosynthesis, redox reaction, and epigenetic pathways. It also highlights the TCA cycle metabolite treatment options for osteoporosis, osteoarthritis, and bone regeneration.

## Figures and Tables

**Table 1:** The putative gene–gene interactions and pathways regulated by key enzymes of the TCA cycle. Bioinformatics clustering was conducted using Cytoscape (http://cytoscape.org, accessed on 23 February 2024).

Name	Gene–Gene Interaction	Biological Activity
*ACOS*	*SOD2, SDHA, IDH3G, IDH3B, IDH3A, IDH2, IDH1, FXN, CS, ACLY*	TCA cycle, pyruvate metabolism, amino acid metabolism, cytosine methylation, cellular energetics, butyrate-induced histone acetylation, mitochondrial complex assembly, glutathione metabolism, fatty acid biosynthesis, lipid metabolism
*IDH*	*ACO1, ACO2, DLST, IDH1, IDH2, IDH3B, IDH3G, OGDH, OGDHL, SUCLA2*	TCA cycle, pyruvate metabolism, cellular energetics, glycolysis and glucogenesis, neuroinflammation and glutamatergic signaling, one-carbon metabolism, glutathione metabolism, histone methylation
*OGDH*	*DLAT, DLD, DLST, IDH1, IDH2, IDH3A, IDH3B, IDH3G, PDHX, SUCLG1*	TCA cycle, pyruvate metabolism, cytosine metabolism, amino acid, VEGF–VEGFRs signaling, angiogenesis, iron metabolism in the placenta, glutathione metabolism, tryptophan metabolism
*SCS*	*ACLY, DLAT, DLST, OGDH, SDHA, SDHB, SDHC, SDHD, SUCLA2 SUCLG2*	TCA cycle, pyruvate metabolism, mitochondrial complex assembly, electron transfer chain in OXPHOS, amino acid metabolism, fatty acid biosynthesis
*SDH*	*FH, NDUFS2, NDUFS8, NDUFV1, SDHAF2, SDHB, SDHC, SDHD, SUCLG1, F5H5T6*	Mitochondrial complex assembly, TCA cycle, electron transfer chain in mitochondrial OXPHOS, TCA cycle in senescence, urea cycle and associated metabolism, cellular energetics
*FH*	*CS GOT2 MDH2 ME1 ME2 ME3 SDHA SDHB SDHC SDHD*	Mitochondrial complex II assembly, amino acid metabolism, TCA cycle, pentose phosphate pathway, NAD metabolism, cellular energetics, glycolysis and glucogenesis
*MDH*	*ACLY, CS, FH, GOT1, GOT1L1, GOT2, IDH2, ME1, ME3, PC*	Butyrate-mediated histone acetylation, TCA cycle in senescence, alanine and aspartate metabolism, urea cycle-associated metabolism, glycolysis and glucogenesis, fatty acid biosynthesis, glutamine metabolism, trans-sulfuration metabolism, pentose phosphate pathway
*CS*	*ACLY, ACO1, ACO2, DLAT, FH, IDH1, IDH2, MDH2, PC, SDHB*	TCA cycle, cellular energetics, cytosine methylation, amino acid metabolism, glycolysis and glucogenesis, urea cycle-associated metabolism, iron metabolism in the placenta

**Table 2:** The putative gene–gene interactions and pathways regulated by FTO and KDMs. Bioinformatics clustering was conducted using Cytoscape (http://cytoscape.org (URL accessed on 23 February 2024)).

Types	Name	Gene–Gene Interaction	Biological Activity
RNA m6N demethylase	*FTO*	*ZMAT3, CLUAP1, CTSA, IRX5, TERF2IP, TKFC, NDRG1, AMFR, PBX3, CKB, NBAS, BCCIP, EEF2, SF3B3, PEPD, LDOC1, GNE, MPPED2, LDHB, CSTF2T*	AMP-activated protein kinase (AMPK) signaling, sterol regulatory element binding protein (SREBP) signaling, FTO obesity variant mechanism, pre-implantation embryo, intercellular component of the RIG receptor pathway, miR-209-3 alteration of the YAP/ECM axis, microRNA regulation of the p53 pathway, Hippo-YAP signaling, urea cycle metabolism
Histone lysine demethylase	*KDM4A*	*ARID1B, CAPZA1, EPHB2, FBXL4, H3C1, H4-16, HARS2, IFNB1, IRF3* *JADE1, KDM4B, KDM4C, KDM4D, KDM4F, NCOR1, PBRM1, RNF168, RNF8, UTP14A*	NIPBL in DNA damage, nucleic acid metabolism, and innate immunity, STING pathway, innate response to dsRNA, TLR4 signaling, and tolerance
Histone lysine demethylase	*KDM5A*	*ARID4A, ARNTL, BPTF, CLOCK, COX20, EMSY, EPB41L5, GATAD1, GLIS1, GPN3, HDAC1 JARID2, MORF4L1, MTF2, PHF12, RSF1, SPAG9, STK4, SUZ12,*	Interactome of polycomb repressive complex (PRC2), circadian rhythm pathway, melatonin metabolism and effects, exercise-induced circadian regulation, valproic acid pathway, hedgehog signaling pathway, sumolyation by RanBP2-regulated transcription repression, transcription co-factor SKI and SKIL protein partners, energy metabolism
Histone lysine demethylase	*KDM6A*	*ASH2L, ATM, DRG1, DYRK1A, FNTA, KLF4, MAP3K2, MSH6, PNPLA4, POU5F1, PROSER1, PUDP, RB1, RBBP5, SIX4, SOX2, SUPT6H, TRAPPC2, WDR5,*	Cell cycle G1/S phase transition, cell cycle G2/M phase transition, cell differentiation, DNA damage response, GABPα/β pathway
Histone lysine demethylase	*KDM7A*	*ALAS2, CDC27, GPCPD1, HSF2BP, KDM2B, KDM3A, KDM4C, KDM6B, KRR1, KRT2, MCL1, PHF13, PHF2, PHF8, PRKD1, RHD, RNF19A, RORA, TDGF1, UHRF1*	Vitamin D-sensitive calcium signaling, coregulation androgen receptor activity, HIF-1α transcription factor network, apoptosis modulation and signaling, microRNA network, heme biosynthesis, nuclear receptors, regulation of apoptosis by parathyroid hormone-related protein, development and heterogeneity of the ILC family, white fat cell differentiation, FBXL10 enhancement of MAP/ERK signaling

## Data Availability

The data are contained within the article.

## References

[1] Leser J.M., Torre O.M., Gould N.R., Guo Q., Buck H.V., Kodama J., Otsuru S., Stains J.P. (2023). Osteoblast-lineage calcium/calmodulin-dependent kinase 2 delta and gamma regulates bone mass and quality. Proc. Natl. Acad. Sci. USA.

[2] Engelmann J., Zarrer J., Gensch V., Riecken K., Berenbrok N., Luu T.V., Beitzen-Heineke A., Vargas-Delgado M.E., Pantel K., Bokemeyer C. (2022). Regulation of bone homeostasis by MERTK and TYRO3. Nat. Commun..

[3] Kim J., Kim B.Y., Lee J.S., Jeong Y.M., Cho H.J., Park E., Kim D., Kim S.S., Kim B.T., Choi Y.J. (2023). UBAP2 plays a role in bone homeostasis through the regulation of osteoblastogenesis and osteoclastogenesis. Nat. Commun..

[4] Vilaca T., Eastell R., Schini M. (2022). Osteoporosis in men. Lancet Diabetes Endocrinol..

[5] Walker M.D., Shane E. (2023). Postmenopausal Osteoporosis. N. Engl. J. Med..

[6] Reid I.R., Billington E.O. (2022). Drug therapy for osteoporosis in older adults. Lancet.

[7] Soós B., Szentpétery Á., Raterman H.G., Lems W.F., Bhattoa H.P., Szekanecz Z. (2022). Effects of targeted therapies on bone in rheumatic and musculoskeletal diseases. Nat. Rev. Rheumatol..

[8] Marques-Carvalho A., Kim H.N., Almeida M. (2023). The role of reactive oxygen species in bone cell physiology and pathophysiology. Bone Rep..

[9] Palmieri E.M., Gonzalez-Cotto M., Baseler W.A., Davies L.C., Ghesquière B., Maio N., Rice C.M., Rouault T.A., Cassel T., Higashi R.M. (2020). Nitric oxide orchestrates metabolic rewiring in M1 macrophages by targeting aconitase 2 and pyruvate dehydrogenase. Nat. Commun..

[10] Piroli G.G., Manuel A.M., McCain R.S., Smith H.H., Ozohanics O., Mellid S., Cox J.H., Cotham W.E., Walla M.D., Cascón A. (2023). Defective function of α-ketoglutarate dehydrogenase exacerbates mitochondrial ATP deficits during complex I deficiency. Redox Biol..

[11] Nanadikar M.S., Vergel Leon A.M., Guo J., van Belle G.J., Jatho A., Philip E.S., Brandner A.F., Böckmann R.A., Shi R., Zieseniss A. (2023). IDH3γ functions as a redox switch regulating mitochondrial energy metabolism and contractility in the heart. Nat. Commun..

[12] Löffler J., Noom A., Ellinghaus A., Dienelt A., Kempa S., Duda G.N. (2023). A comprehensive molecular profiling approach reveals metabolic alterations that steer bone tissue regeneration. Commun. Biol..

[13] Madhu V., Hernandaz-Meadows M., Coleman A., Sao K., Inguito K., Haslam O., Boneski P.K., Sesaki H., Collins J.A., Risbud M.V. (2024). OPA1 protects intervertebral disc and knee joint health in aged mice by maintaining the structure and metabolic functions of mitochondria. bioRxiv.

[14] Wu Y.L., Lin Z.J., Li C.C., Lin X., Shan S.K., Guo B., Zheng M.H., Li F., Yuan L.Q., Li Z.H. (2023). Epigenetic regulation in metabolic diseases: Mechanisms and advances in clinical study. Signal Transduct. Target Ther..

[15] Roig-Soriano J., Griñán-Ferré C., Espinosa-Parrilla J.F., Abraham C.R., Bosch A., Pallàs M., Chillón M. (2022). AAV-mediated expression of secreted and transmembrane αKlotho isoforms rescues relevant aging hallmarks in senescent SAMP8 mice. Aging Cell.

[16] Yang C., Dong Z., Ling Z., Chen Y. (2022). The crucial mechanism and therapeutic implication of RNA methylation in bone pathophysiology. Aging Res. Rev..

[17] Yin B., Yu F., Wang C., Li B., Liu M., Ye L. (2019). Epigenetic control of mesenchymal stem cell fate decision via histone methyltransferase Ash1l. Stem Cells.

[18] Liu X., Si W., He L., Yang J., Peng Y., Ren J., Liu X., Jin T., Yu H., Zhang Z. (2021). The existence of a nonclassical TCA cycle in the nucleus that wires the metabolic-epigenetic circuitry. Signal Transduct. Target Ther..

[19] Jaccard A., Wyss T., Maldonado-Pérez N., Rath J.A., Bevilacqua A., Peng J.J., Lepez A., Von Gunten C., Franco F., Kao K.C. (2023). Reductive carboxylation epigenetically instructs T cell differentiation. Nature.

[20] Ly C.H., Lynch G.S., Ryall J.G. (2020). A metabolic roadmap for somatic stem cell fate. Cell Metab..

[21] Bonnay F., Veloso A., Steinmann V., Köcher T., Abdusselamoglu M.D., Bajaj S., Rivelles E., Landskron L., Esterbauer H., Zinzen R.P. (2020). Oxidative metabolism drives immortalization of neural stem cells during tumorigenesis. Cell.

[22] Rohatgi N., Zou W., Li Y., Cho K., Collins P.L., Tycksen E., Pandey G., DeSelm C.J., Patti G.J., Dey A. (2023). BAP1 promotes osteoclast function by metabolic reprogramming. Nat. Commun..

[23] Wu X., Dai H., Yu S., Zhao Y., Long Y., Li W., Tu J. (2021). Citrate regulates extracellular matrix mineralization during osteoblast differentiation in vitro. J. Inorg. Biochem..

[24] Dirckx N., Zhang Q., Chu E.Y., Tower R.J., Li Z., Guo S., Yuan S., Khare P.A., Zhang C., Verardo A. (2022). A specialized metabolic pathway partitions citrate in hydroxyapatite to impact mineralization of bones and teeth. Proc. Natl. Acad. Sci. USA.

[25] Villaseñor A., Aedo-Martín D., Obeso D., Erjavec I., Rodríguez-Coira J., Buendía I., Ardura J.A., Barbas C., Gortazar A.R. (2019). Metabolomics reveals citric acid secretion in mechanically-stimulated osteocytes is inhibited by high glucose. Sci. Rep..

[26] Chen H., Wang Y., Dai H., Tian X., Cui Z.K., Chen Z., Hu L., Song Q., Liu A., Zhang Z. (2018). Bone and plasma citrate is reduced in osteoporosis. Bone.

[27] Wei J., Yang Y., Guo D., Xu S., Huang H., Zhang D., Xie J., Zhou X. (2022). Osteoblasts induce glucose-derived ATP perturbations in chondrocytes through noncontact communication. Acta Biochim. Biophys. Sin..

[28] Monteiro C., Ferreira de Oliveira J.M.P., Pinho F., Bastos V., Oliveira H., Peixoto F., Santos C. (2018). Biochemical and transcriptional analyses of cadmium-induced mitochondrial dysfunction and oxidative stress in human osteoblasts. J. Toxicol. Environ. Health A.

[29] Ni S., Yuan Y., Qian Z., Zhong Z., Lv T., Kuang Y., Yu B. (2021). Hypoxia inhibits RANKL-induced ferritinophagy and protects osteoclasts from ferroptosis. Free Radic. Biol. Med..

[30] Bonadonna M., Altamura S., Tybl E., Palais G., Qatato M., Polycarpou-Schwarz M., Schneider M., Kalk C., Rüdiger W., Ertl A. (2022). Iron regulatory protein (IRP)-mediated iron homeostasis is critical for neutrophil development and differentiation in the bone marrow. Sci. Adv..

[31] Chen C., Zhang Z., Liu C., Sun P., Liu P., Li X. (2024). ABCG2 is an itaconate exporter that limits antibacterial innate immunity by alleviating TFEB-dependent lysosomal biogenesis. Cell Metab..

[32] Song J., Zhang Y., Frieler R.A., Andren A., Wood S., Tyrrell D.J., Sajjakulnukit P., Deng J.C., Lyssiotis C.A., Mortensen R.M. (2023). Itaconate suppresses atherosclerosis by activating a Nrf2-dependent antiinflammatory response in macrophages in mice. J. Clin. Investig..

[33] Ramalho T., Assis P.A., Ojelabi O., Tan L., Carvalho B., Gardinassi L., Campos O., Lorenzi P.L., Fitzgerald K.A., Haynes C. (2024). Itaconate impairs immune control of Plasmodium by enhancing mtDNA-mediated PD-L1 expression in monocyte-derived dendritic cells. Cell Metab..

[34] Maassen S., Coenen B., Ioannidis M., Harber K., Grijpstra P., Van den Bossche J., van den Bogaart G. (2023). Itaconate promotes a wound resolving phenotype in pro-inflammatory macrophages. Redox Biol..

[35] Lippross S., Beckmann R., Streubesand N., Ayub F., Tohidnezhad M., Campbell G., Kan Y.W., Horst F., Sönmez T.T., Varoga D. (2014). Nrf2 deficiency impairs fracture healing in mice. Calcif. Tissue Int..

[36] Sun X., Zhang B., Pan X., Huang H., Xie Z., Ma Y., Hu B., Wang J., Chen Z., Shi P. (2019). Octyl itaconate inhibits osteoclastogenesis by suppressing Hrd1 and activating Nrf2 signaling. FASEB J..

[37] Zheng Y., Chen Z., She C., Lin Y., Hong Y., Shi L., Zhang Y., Cao P., Xu X. (2020). Four-octyl itaconate activates Nrf2 cascade to protect osteoblasts from hydrogen peroxide-induced oxidative injury. Cell Death Dis..

[38] Kubo Y., Wruck C.J., Fragoulis A., Drescher W., Pape H.C., Lichte P., Fischer H., Tohidnezhad M., Hildebrand F., Pufe T. (2019). Role of Nrf2 in fracture healing: Clinical aspects of oxidative stress. Calcif. Tissue Int..

[39] Chae U., Park N.R., Kim E.S., Choi J.Y., Yim M., Lee H.S., Lee S.R., Lee S., Park J.W., Lee D.S. (2018). IDH2-deficient mice develop spinal deformities with aging. Physiol. Res..

[40] Lee S.H., Lee S.H., Lee J.H., Park J.W., Kim J.E. (2019). IDH2 deficiency increases bone mass with reduced osteoclastogenesis by limiting RANKL expression in osteoblasts. Bone.

[41] Wang Y., Deng P., Liu Y., Wu Y., Chen Y., Guo Y., Zhang S., Zheng X., Zhou L., Liu W. (2020). Alpha-ketoglutarate ameliorates age-related osteoporosis via regulating histone methylations. Nat. Commun..

[42] Liu L., Zhang W., Liu T., Tan Y., Chen C., Zhao J., Geng H., Ma C. (2023). The physiological metabolite α-ketoglutarate ameliorates osteoarthritis by regulating mitophagy and oxidative stress. Redox Biol..

[43] Stegen S., Moermans K., Stockmans I., Thienpont B., Carmeliet G. (2024). The serine synthesis pathway drives osteoclast differentiation through epigenetic regulation of NFATc1 expression. Nat. Metab..

[44] Lee S., Kim H.S., Kim M.J., Min K.Y., Choi W.S., You J.S. (2021). Glutamine metabolite α-ketoglutarate acts as an epigenetic co-factor to interfere with osteoclast differentiation. Bone.

[45] Cai W., Zhang J., Yu Y., Ni Y., Wei Y., Cheng Y., Han L., Xiao L., Ma X., Wei H. (2023). Mitochondrial transfer regulates cell fate through metabolic remodeling in osteoporosis. Adv. Sci..

[46] Guo Y., Xu F., Thomas S.C., Zhang Y., Paul B., Sakilam S., Chae S., Li P., Almeter C., Kamer A.R. (2022). Targeting the succinate receptor effectively inhibits periodontitis. Cell Rep..

[47] Lee W.C., Ji X., Nissim I., Long F. (2020). Malic enzyme couples mitochondria with aerobic glycolysis in osteoblasts. Cell Rep..

[48] Michaletti A., Gioia M., Tarantino U., Zolla L. (2017). Effects of microgravity on osteoblast mitochondria: A proteomic and metabolomics profile. Sci. Rep..

[49] Sánchez-de-Diego C., Pedrazza L., Pimenta-Lopes C., Martinez-Martinez A., Dahdah N., Valer J.A., Garcia-Roves P., Rosa J.L., Ventura F. (2021). NRF2 function in osteocytes is required for bone homeostasis and drives osteocytic gene expression. Redox Biol..

[50] Jaramillo J., Taylor C., McCarley R., Berger M., Busse E., Sammarco M.C. (2023). Oxaloacetate enhances and accelerates regeneration in young mice by promoting proliferation and mineralization. Front. Cell Dev. Biol..

[51] Tower R.J., Busse E., Jaramillo J., Lacey M., Hoffseth K., Guntur A.R., Simkin J., Sammarco M.C. (2022). Spatial transcriptomics reveals metabolic changes underly age-dependent declines in digit regeneration. Elife.

[52] Guo Q., Kang H., Wang J., Dong Y., Peng R., Zhao H., Wu W., Guan H., Li F. (2021). Inhibition of ACLY leads to suppression of osteoclast differentiation and function via regulation of histone acetylation. J. Bone Miner. Res..

[53] Pouikli A., Maleszewska M., Parekh S., Yang M., Nikopoulou C., Bonfiglio J.J., Mylonas C., Sandoval T., Schumacher A.L., Hinze Y. (2022). Hypoxia promotes osteogenesis by facilitating acetyl-CoA-mediated mitochondrial-nuclear communication. EMBO J..

[54] Stegen S., Rinaldi G., Loopmans S., Stockmans I., Moermans K., Thienpont B., Fendt S.M., Carmeliet P., Carmeliet G. (2020). Glutamine metabolism controls chondrocyte identity and function. Dev. Cell.

[55] Watanabe K., Iida M., Harada S., Kato S., Kuwabara K., Kurihara A., Takeuchi A., Sugiyama D., Okamura T., Suzuki A. (2022). Metabolic profiling of charged metabolites in association with menopausal status in Japanese community-dwelling midlife women: Tsuruoka Metabolomic Cohort Study. Maturitas.

[56] Yu Y., Newman H., Shen L., Sharma D., Hu G., Mirando A.J., Zhang H., Knudsen E., Zhang G.F., Hilton M.J. (2019). Glutamine metabolism regulates proliferation and lineage allocation in skeletal stem cells. Cell Metab..

[57] Liu X., Yan Z., Cai J., Wang D., Yang Y., Ding Y., Shao X., Hao X., Luo E., Guo X.E. (2023). Glucose- and glutamine-dependent bioenergetics sensitize bone mechanoresponse after unloading by modulating osteocyte calcium dynamics. J. Clin. Investig..

[58] Guo Q., Zhao H., Dong Z., Cheng H., Zhu M., Fang Z. (2024). Inhibiting glutaminase exerts opposite effects on ovariectomy-induced and age-related reductions in murine bone mass. Aging Dis..

[59] Peng R., Dong Y., Zheng M., Kang H., Wang P., Zhu M., Song K., Wu W., Li F. (2024). IL-17 promotes osteoclast-induced bone loss by regulating glutamine-dependent energy metabolism. Cell Death Dis..

[60] Huang X., Lan Y., Shen J., Zhao X., Zhou Y., Wu W., Mao J., Wu Y., Xie Z., Chen Z. (2024). M2 macrophages secrete glutamate-containing extracellular vesicles to alleviate osteoporosis by reshaping osteoclast precursor fate. Mol. Ther..

[61] Go M., Shin E., Jang S.Y., Nam M., Hwang G.S., Lee S.Y. (2022). BCAT1 promotes osteoclast maturation by regulating branched-chain amino acid metabolism. Exp. Mol. Med..

[62] Vujic A., Koo A.N.M., Prag H.A., Krieg T. (2021). Mitochondrial redox and TCA cycle metabolite signaling in the heart. Free Radic. Biol. Med..

[63] Mansilla S., Tórtora V., Pignataro F., Sastre S., Castro I., Chiribao M.L., Robello C., Zeida A., Santos J., Castro L. (2023). Redox sensitive human mitochondrial aconitase and its interaction with frataxin: In vitro and in silico studies confirm that it takes two to tango. Free Radic. Biol. Med..

[64] Chalifoux O., Faerman B., Mailloux R.J. (2023). Mitochondrial hydrogen peroxide production by pyruvate dehydrogenase and α-ketoglutarate dehydrogenase in oxidative eustress and oxidative distress. J. Biol. Chem..

[65] Noh M.R., Kong M.J., Han S.J., Kim J.I., Park K.M. (2020). Isocitrate dehydrogenase 2 deficiency aggravates prolonged high-fat diet intake-induced hypertension. Redox Biol..

[66] Lee J.H., Go Y., Kim D.Y., Lee S.H., Kim O.H., Jeon Y.H., Kwon T.K., Bae J.H., Song D.K., Rhyu I.J. (2020). Isocitrate dehydrogenase 2 protects mice from high-fat diet-induced metabolic stress by limiting oxidative damage to the mitochondria from brown adipose tissue. Exp. Mol. Med..

[67] Ryan D.G., Yang M., Prag H.A., Blanco G.R., Nikitopoulou E., Segarra-Mondejar M., Powell C.A., Young T., Burger N., Miljkovic J.L. (2021). Disruption of the TCA cycle reveals an ATF4-dependent integration of redox and amino acid metabolism. Elife.

[68] Altea-Manzano P., Vandekeere A., Edwards-Hicks J., Roldan M., Abraham E., Lleshi X., Guerrieri A.N., Berardi D., Wills J., Junior J.M. (2022). Reversal of mitochondrial malate dehydrogenase 2 enables anaplerosis via redox rescue in respiration-deficient cells. Mol. Cell..

[69] Stegen S., Carmeliet G. (2024). Metabolic regulation of skeletal cell fate and function. Nat. Rev. Endocrinol..

[70] Tao H., Li X., Wang Q., Yu L., Yang P., Chen W., Yang X., Zhou J., Geng D. (2024). Redox signaling and antioxidant defense in osteoclasts. Free Radic. Biol. Med..

[71] Kubo Y., Beckmann R., Fragoulis A., Conrads C., Pavanram P., Nebelung S., Wolf M., Wruck C.J., Jahr H., Pufe T. (2022). Nrf2/ARE signaling directly regulates SOX9 to potentially alter age-dependent cartilage degeneration. Antioxidants..

[72] Chakrabarty R.P., Chandel N.S. (2021). Mitochondria as signaling organelles control mammalian stem cell fate. Cell Stem Cell.

[73] Li W., Long Q., Wu H., Zhou Y., Duan L., Yuan H., Ding Y., Huang Y., Wu Y., Huang J. (2022). Nuclear localization of mitochondrial TCA cycle enzymes modulates pluripotency via histone acetylation. Nat. Commun..

[74] Liu Q., Zhu F., Liu X., Lu Y., Yao K., Tian N., Tong L., Figge D.A., Wang X., Han Y. (2022). Non-oxidative pentose phosphate pathway controls regulatory T cell function by integrating metabolism and epigenetics. Nat. Metab..

[75] Kafkia E., Andres-Pons A., Ganter K., Seiler M., Smith T.S., Andrejeva A., Jouhten P., Pereira F., Franco C., Kuroshchenkova A. (2022). Operation of a TCA cycle subnetwork in the mammalian nucleus. Sci. Adv..

[76] Tournaire G., Loopmans S., Stegen S., Rinaldi G., Eelen G., Torrekens S., Moermans K., Carmeliet P., Ghesquière B., Thienpont B. (2022). Skeletal progenitors preserve proliferation and self-renewal upon inhibition of mitochondrial respiration by rerouting the TCA cycle. Cell Rep..

[77] Michalak E.M., Burr M.L., Bannister A.J., Dawson M.A. (2019). The roles of DNA, RNA and histone methylation in ageing and cancer. Nat. Rev. Mol. Cell Biol..

[78] Chen Y.C., Koutelou E., Dent S.Y.R. (2022). Now open: Evolving insights to the roles of lysine acetylation in chromatin organization and function. Mol. Cell..

[79] Traube F.R., Özdemir D., Sahin H., Scheel C., Glück A.F., Geserich A.S., Oganesian S., Kostidis S., Iwan K., Rahimoff R. (2021). Redirected nuclear glutamate dehydrogenase supplies Tet3 with α-ketoglutarate in neurons. Nat. Commun..

[80] Ming-Chin Lee K., Achuthan A.A., De Souza D.P., Lupancu T.J., Binger K.J., Lee M.K.S., Xu Y., McConville M.J., de Weerd N.A., Dragoljevic D. (2022). Type I interferon antagonism of the JMJD3-IRF4 pathway modulates macrophage activation and polarization. Cell Rep..

[81] Wei J., Yu X., Yang L., Liu X., Gao B., Huang B., Dou X., Liu J., Zou Z., Cui X.L. (2022). FTO mediates LINE1 m6A demethylation and chromatin regulation in mESCs and mouse development. Science.

[82] Shvedunova M., Akhtar A. (2022). Modulation of cellular processes by histone and non-histone protein acetylation. Nat. Rev. Mol. Cell Biol..

[83] Anmangandla A., Ren Y., Fu Q., Zhang S., Lin H. (2022). The Acyl-CoA specificity of human lysine acetyltransferase KAT2A. Biochemistry.

[84] Zhang L., Zhu K., Xu J., Chen X., Sheng C., Zhang D., Yang Y., Sun L., Zhao H., Wang X. (2023). Acetyltransferases CBP/p300 control transcriptional switch of β-Catenin and Stat1 promoting osteoblast differentiation. J. Bone Miner. Res..

[85] Abe Y., Kofman E.R., Ouyang Z., Cruz-Becerra G., Spann N.J., Seidman J.S., Troutman T.D., Stender J.D., Taylor H., Fan W. (2024). A TLR4/TRAF6-dependent signaling pathway mediates NCoR coactivator complex formation for inflammatory gene activation. Proc. Natl. Acad. Sci. USA.

[86] Tsukasaki M., Huynh N.C., Okamoto K., Muro R., Terashima A., Kurikawa Y., Komatsu N., Pluemsakunthai W., Nitta T., Abe T. (2020). Stepwise cell fate decision pathways during osteoclastogenesis at single-cell resolution. Nat. Metab..

[87] Pezoa S.A., Artinger K.B., Niswander L.A. (2020). GCN5 acetylation is required for craniofacial chondrocyte maturation. Dev. Biol..

[88] Chen J., Liu D., Chen B., Yang Y., Zhu H., Li D., Liu K., Zhu L., Liu H., Li M. (2023). The histone acetyltransferase Mof regulates Runx2 and Osterix for osteoblast differentiation. Cell Tissue Res..

[89] Wu Y., Zhang H., Tang M., Guo C., Deng A., Li J., Wang Y., Xiao L., Yang G. (2020). High methylation of lysine acetyltransferase 6B is associated with the Cobb angle in patients with congenital scoliosis. J. Transl. Med..

[90] Wang L., You X., Ruan D., Shao R., Dai H.Q., Shen W., Xu G.L., Liu W., Zou W. (2022). TET enzymes regulate skeletal development through increasing chromatin accessibility of RUNX2 target genes. Nat. Commun..

[91] Kubo Y., Gonzalez J.A.H., Beckmann R., Weiler M., Pahlavani H., Saldivar M.C., Szymanski K., Rosenhain S., Fragoulis A., Leeflang S. (2022). Nuclear factor erythroid 2-related factor 2 (Nrf2) deficiency causes age-dependent progression of female osteoporosis. BMC Musculoskelet. Disord..

[92] Thaler R., Khani F., Sturmlechner I., Dehghani S.S., Denbeigh J.M., Zhou X., Pichurin O., Dudakovicm A., Jerez S.S., Zhong J. (2022). Vitamin C epigenetically controls osteogenesis and bone mineralization. Nat. Commun..

[93] Dusadeemeelap C., Rojasawasthien T., Matsubara T., Kokabu S., Addison W.N. (2022). Inhibition of TET-mediated DNA demethylation suppresses osteoblast differentiation. FASEB J..

[94] Cakouros D., Hemming S., Gronthos K., Liu R., Zannettino A., Shi S., Gronthos S. (2019). Specific functions of TET1 and TET2 in regulating mesenchymal cell lineage determination. Epigenetics Chromatin..

[95] Yang C., Tao H., Zhang H., Xia Y., Bai J., Ge G., Li W., Zhang W., Xiao L., Xu Y. (2022). TET2 regulates osteoclastogenesis by modulating autophagy in OVX-induced bone loss. Autophagy.

[96] Liang J., Sun J., Zhang W., Wang X., Xu Y., Peng Y., Zhang L., Xiong W., Liu Y., Liu H. (2023). Novel insights into the roles of N6-methyladenosine (m6A) modification and autophagy in human diseases. Int. J. Biol. Sci..

[97] Chen L.S., Zhang M., Chen P., Xiong X.F., Liu P.Q., Wang H.B., Wang J.J., Shen J. (2022). The m6A demethylase FTO promotes the osteogenesis of mesenchymal stem cells by downregulating PPARG. Acta Pharmacol. Sin..

[98] Zhang Q., Riddle R.C., Yang Q., Rosen C.R., Guttridge D.C., Dirckx N., Faugere M.C., Farber C.R., Clemens T.L. (2019). The RNA demethylase FTO is required for maintenance of bone mass and functions to protect osteoblasts from genotoxic damage. Proc. Natl. Acad. Sci. USA.

[99] Zhuang J., Ning H., Wang M., Zhao W., Jing Y., Liu X., Zu J., Kong P., Wang X., Sun C. (2021). Downregulated fat mass and obesity-associated protein inhibits bone resorption and osteoclastogenesis by nuclear factor-kappa B inactivation. Cell Signal.

[100] Li Z., Wang P., Li J., Xie Z., Cen S., Li M., Liu W., Ye G., Zheng G., Ma M. (2021). The N6-methyladenosine demethylase ALKBH5 negatively regulates the osteogenic differentiation of mesenchymal stem cells through PRMT6. Cell Death Dis..

[101] Feng L., Fan Y., Zhou J., Li S., Zhang X. (2021). The RNA demethylase ALKBH5 promotes osteoblast differentiation by modulating Runx2 mRNA stability. FEBS Lett..

[102] Qi Q., Wang Y., Wang X., Yang J., Xie Y., Zhou J., Li X., Wang B. (2020). Histone demethylase KDM4A regulates adipogenic and osteogenic differentiation via epigenetic regulation of C/EBPα and canonical Wnt signaling. Cell. Mol. Life Sci..

[103] Deng P., Yuan Q., Cheng Y., Li J., Liu Z., Liu Y., Li Y., Su T., Wang J., Salvo M.E. (2021). Loss of KDM4B exacerbates bone-fat imbalance and mesenchymal stromal cell exhaustion in skeletal aging. Cell Stem Cell.

[104] Yi S.J., Jang Y.J., Kim H.J., Lee K., Lee H., Kim Y., Kim J., Hwang S.Y., Song J.S., Okada H. (2021). The KDM4B-CCAR1-MED1 axis is a critical regulator of osteoclast differentiation and bone homeostasis. Bone Res..

[105] Lu T., Forgetta V., Zhou S., Richards J.B., Greenwood C.M. (2023). Identifying rare genetic determinants for improved polygenic risk prediction of bone mineral density and fracture risk. J. Bone Miner. Res..

[106] Wang C., Wang J., Li J., Hu G., Shan S., Li Q., Zhang X. (2016). KDM5A controls bone morphogenic protein 2-induced osteogenic differentiation of bone mesenchymal stem cells during osteoporosis. Cell Death Dis..

[107] Liu H., Zhai L., Liu Y., Lu D., Vander Ark A., Yang T., Krawczyk C.M. (2023). The histone demethylase KDM5C controls female bone mass by promoting energy metabolism in osteoclasts. Sci. Adv..

[108] Gao C.W., Lin W., Riddle R.C., Kushwaha P., Boukas L., Björnsson H.T., Hansen K.D., Fahrner J.A. (2024). A mouse model of Weaver syndrome displays overgrowth and excess osteogenesis reversible with KDM6A/6B inhibition. JCI Insight.

[109] Pribadi C., Cakouros D., Camp E., Anderson P., Gronthos S. (2023). KDM6A-mediated regulation of cranial frontal bone suture fusion in mice is sex dependent. Stem Cells Dev..

[110] Liu Z., Lee H.L., Suh J.S., Deng P., Lee C.R., Bezouglaia O., Mirnia M., Chen V., Zhou M., Cui Z.K. (2022). The ERα/KDM6B regulatory axis modulates osteogenic differentiation in human mesenchymal stem cells. Bone Res..

[111] Behera J., Ison J., Voor M.J., Tyagi N. (2021). Probiotics stimulate bone formation in obese mice via histone methylations. Theranostics.

[112] Shan L., Yang X., Liao X., Yang Z., Zhou J., Li X., Wang B. (2024). Histone demethylase KDM7A regulates bone homeostasis through balancing osteoblast and osteoclast differentiation. Cell Death Dis..

[113] Assi R., Cherifi C., Cornelis F.M.F., Zhou Q., Storms L., Pazmino S., Coutinho de Almeida R., Meulenbelt I., Lories R.J., Monteagudo S. (2023). Inhibition of KDM7A/B histone demethylases restores H3K79 methylation and protects against osteoarthritis. Ann. Rheum. Dis..

[114] Cheng C., Xing Z., Hu Q., Kong N., Liao C., Xu S., Zhang J., Kang F., Zhu X. (2024). A bone-targeting near-infrared luminescence nanocarrier facilitates alpha-ketoglutarate efficacy enhancement for osteoporosis therapy. Acta Biomater..

[115] Wang Z., Hu J., Faber J., Miszuk J., Sun H. (2022). Locally delivered metabolite derivative promotes bone regeneration in aged mice. ACS Appl. Bio Mater..

[116] Chen X., Li C., Cao X., Jia X., Chen X., Wang Z., Xu W., Dai F., Zhang S. (2022). Mitochondria-targeted supramolecular coordination container encapsulated with exogenous itaconate for synergistic therapy of joint inflammation. Theranostics.

[117] Ni L., Lin Z., Hu S., Shi Y., Jiang Z., Zhao J., Zhou Y., Wu Y., Tian N., Sun L. (2022). Itaconate attenuates osteoarthritis by inhibiting STING/NF-κB axis in chondrocytes and promoting M2 polarization in macrophages. Biochem. Pharmacol..

